# Relationship Between Bio-Climatic and Milk Composition Data of Dairy Sheep Farms: Comparison Between THI and Multivariate Weather Index

**DOI:** 10.3390/ani15040533

**Published:** 2025-02-13

**Authors:** Rita Marras, Alfredo Pauciullo, Alberto Cesarani, Antonio Natale, Paolo Oppia, Nicolò P. P. Macciotta, Giustino Gaspa

**Affiliations:** 1 Department of Agriculture, University of Sassari, 07100 Sassari, Italy; ritamarras@hotmail.com (R.M.); acesarani@uniss.it (A.C.); macciott@uniss.it (N.P.P.M.); 2 Department of Agricultural, Forest and Food Science, University of Torino, 10095 Grugliasco, Italy; 3 National Research Council of Italy, Institute of Animal Production in the Mediterranean Environment, 80055 Portici, Italy; 4 Department of Animal and Dairy Science, University of Georgia, Athens, GA 30602, USA; 5 Associazione Regionale Allevatori della Sardegna (ARAS), 09100 Cagliari, Italy

**Keywords:** climate change, heat stress, dairy sheep, milk composition, multivariate statistics

## Abstract

Milk yield and its composition show individual variation due to the effects of the environment. Previous studies suggest that meteorological variables exert negative effects on milk yield and composition, especially during summer. This study aimed to examine the effects of meteorological variables on bulk milk composition in the Sardinian sheep production system. In this work, a total of 218,170 records belonging to 4562 dairy sheep farms were merged with the meteorological data provided by 60 meteorological stations located on Sardinia Island (Italy). Milk composition in the late spring and summer recorded during a 5-year period was used to evaluate the impact of climate exposure on bulk milk traits. The milk quality was analyzed using a linear mixed model that included the fixed effects of the year of sampling, the flock size, the temperature humidity index (THI) and the random effect of the flock. The variability of milk composition explained by flock and management ranged from 30 to 64%. The flock size exerted a significant effect on milk composition: large flocks characterized by advanced management and feeding techniques resulted in higher milk quality (e.g., higher protein and fat, lower lactose) compared to traditionally managed small flocks. The impact of THI on milk composition was statistically significant across different milk quality traits (*p* < 0.001); the effect of thermal stress varied according to the month of lactation. For instance, milk fat content in May increased by +0.4% for THI > 76. In June, no relevant differences were observed, whereas a decrease in fat percentage was observed in July as THI values increased (up to −0.5% for THI > 76). While somatic cell counts remained relatively stable across different conditions, total bacterial count showed greater seasonal variability, peaking during warmer periods. In addition, using factor analysis, we developed a multivariate meteorological index (MMI), which explained 51% of the variance of the original meteorological data. MMI was highly correlated with THI (r = 0.75). The same linear mixed model applied for modeling THI was used to assess the effect of MMI on milk traits. Fat, protein fractions and lactose showed significant variation across MMI classes (*p*-value < 0.001) in the same direction as those based on THI. Overall, our findings underscore the impact of both flock size and environmental conditions on milk quality, with heat stress and traditional versus modern management practices leading to measurable differences in milk traits.

## Simple Summary

This study explores how heat affects sheep milk production in Mediterranean climatic conditions. This research examined milk composition changes during late spring and summer. The bulk milk compositions from about 4500 farms over a five-year period were used for this study. Weather data from local stations were matched with milk quality data of farms near the weather stations. To understand the impact of heat stress on milk composition, we used both a temperature–humidity index and a multivariate meteorological index that combined temperature, relative humidity, precipitation and wind speed. The impact of bio-meteorological indexes on milk composition was statistically significant across different bulk milk quality traits. These findings point out how temperature and humidity affect milk quality, and that heat exerts negative effects on milk composition.

## 1. Introduction

Weather conditions impact animals and exert a strong influence on animal production, reproduction and health [1]. Environmental stressors such as temperature, relative humidity and solar radiation can adversely affect animal performance and reproduction, with direct and indirect effects [2], leading to economic losses [3,4,5,6,7]. Sheep and goats have a wide geographical distribution and an economic impact worldwide; therefore, knowledge of how their performance is influenced in response to heat stress is essential. Different studies have been conducted on heat tolerance in cattle [8,9,10,11] and small ruminants [12,13,14,15,16,17], but the effect of heat stress on dairy sheep is poorly investigated. Sheep and goat milk globally represent only 1.3% and 1.9% of the world’s total milk, respectively (www.faostat.org, accessed on 6 December 2024). Despite these global figures, in the Mediterranean and (semi)arid areas, dairy sheep farming is vital for the local dairy industry [18,19], and the effects of an adverse climate could further exacerbate the economic marginality of the dairy sheep sector. The unfavorable effects of thermal stress on dairy sheep have been examined, with little agreement on defining a threshold for thermal neutrality [16,20,21]. Furthermore, heat tolerance is negatively correlated with milk yield, and the reduction in performance is greatest in high-yielding sheep and goats [13,22,23,24].

Temperatures in the Mediterranean area can often exceed the sheep thermoneutral zone, impairing sheep productivity. Over the last twenty years, the global increasing trend in temperatures has been confirmed by several international agencies (https://www.copernicus.eu/en, https://www.ipcc.ch/report/sixth-assessment-report-cycle/; accessed on 6 September 2024), and its effects on local climatic conditions are mostly unpredictable. The effects on many livestock species have been widely investigated [1,25,26,27]. Outside the thermal neutral zone, productive and reproductive traits are generally impaired [12], and heat tolerance changes across species and breeds within species. Individual differences in heat tolerance regarding animal production can be partially ascribed to genetic effects and adaptation strategies [12,13,24,28]. The pastoral livestock systems of southern Europe are mostly exposed to high temperatures during the summer. As far as dairy sheep are concerned, critical temperatures and their effects on milk productivity are rather variable. For instance, Sarda ewes are in the thermal neutrality zone with reference to milk yield when exposed to minimum temperatures between 9 and 12 °C [16,20,29] and maximum temperatures between 15 and 24 °C [29]. Instead, for Spanish Manchega sheep, the optimum ranges from −1 to 10.2 °C and between 19.1 and 29.6 °C for minimum and maximum temperatures, respectively [24]. The most common bio-meteorological index used for farm animals is the temperature–humidity index (THI), which combines temperature and humidity. Several studies on dairy cattle—which are highly sensitive to heat waves—report different THI measures and thresholds: (i) THI_MAX_, which combines daily maximum temperature and minimum humidity in order to represent the worst environmental conditions in the diurnal hours; (ii) THI_MIN_, computed using daily minimum temperature and maximum humidity, which refers to day–night conditions; and (iii) THI_AVG_, which uses daily average temperature and humidity [30,31,32].

The current study investigated the effects of environmental and meteorological conditions in late spring and summer on sheep milk quality. Sardinian dairy farms were used as an example of southern Europe’s farming system exposed to a hot environment. To evaluate the effects of THI on bulk milk composition traits, the variation in sheep milk quality was assessed at the farm level, analyzing a large sample of dairy farms. Despite being restricted to the farm level, the results are important regarding farm profitability since sheep milk is almost entirely processed into cheese. Two approaches were adopted to address the research question. The first was aimed at estimating the effect of THI on bulk milk composition 1 day (or 2 days) before milk sampling; these estimates were adjusted for the other known environmental factors. The second objective was to test alternative meteorological indexes that combined all the available meteorological variables (temperatures, humidity, wind speed, rainfall and solar radiation) through a multivariate statistics approach.

## 2. Materials and Methods

### 2.1. Bulk Milk Composition Data

The analyzed data came from 4562 sheep farms for a total of 218,170 monthly bulk milk records. The dataset, provided by the breeder association, included the geographical, structural (i.e., no. hectares, flock sizes, facilities and equipment, organic or conventional farming system, etc.), productive (total annual milk yield per farm) and milk composition data collected during a period of 5 years from 2003 to 2008. Briefly, the bulk milk samples were analyzed for milk fat, protein, casein and lactose contents (g/100 mL milk for all traits, approximate by %) determined by MilkoScan^TM^ 6000 (Foss, Hilleroed, Danmark); SCC via automated flow cytometry devices using the Fossomatic^TM^ (Foss, Hilleroed, Denmark); and total bacterial load using Bactoscan^TM^ FC (Foss, Hilleroed, Denmark). The logarithmic transformation (log_10_) of total bacterial count (log_TBC_) and somatic cell count (log_SCC_) was used to normalize the distributions of somatic cells and bacterial counts. The yearly distribution of milk data records available per flock is reported in Supplementary Figure S1. The focus on bulk milk data, instead of individual milk, was motivated by the size and representativeness of the Sardinian farms, according to the objective of tracing the effect of thermal stress events at the farm level.

### 2.2. Meteorological Data

The meteorological data were derived from the weather stations closest to the 4562 farms (<50 km from farms’ gates); they were supplied by the Sardinian meteorological network (60 stations evenly spread throughout the regional territory) of the Regional Agency of Environment Protection (ARPAS, https://www.sar.sardegna.it/, accessed on 6 September 2024). The 50 km criterium—the maximum distance between the meteorological stations and farms—was used to represent the meteorological conditions near the farms. The ideal situation is to have the weather data from the stations located on the farm; however, a proximity criterium is widely used in the literature when large datasets are analyzed [13,30,33]. For each farm, milk composition data were merged with meteorological variables from the nearest stations (Supplementary Table S1 and Figure S2). The final number of weather records ranged from 20,461 to 61,499 due to the presence of missing data in different variables. Weather data consisted of daily maximum, minimum and average temperatures (min, max, and avg, °C); daily wind speed (m/s. max and average); daily rainfall (mm); and relative humidity (RH, %), referring to the same five-year period of milk sample collection (Supplementary Figure S3). The THI was calculated according to Kliber (1964) [16], which was used for Sarda breeds in Mediterranean conditions in previous studies:

(1) $$\mathrm{THI}\;=1.8T_{\mathrm{db}}–\;\left(1-\frac{\mathrm{RH}\;}{100}\right)\;\cdot \;\left(T_{\mathrm{db}}-14.3\right)+32$$

where Tdb = air temperature (°C) and RH = relative humidity. Equation (1) was applied to compute the maximum (THI_MAX_) and average THI (THI_AVG_) by combining values of daily temperatures and humidity. Meteorological data referred to the whole island from late spring to autumn (from May to November). The dataset used for the statistical analyses was assembled selecting data records that presented both milk quality and bio-climatic variables for each sampling date. Due to the strict seasonality of Sarda dairy sheep [19], the data records from August to November were discarded (due to low data density). Furthermore, farms with fewer than 4 milk samples per year were excluded. Additionally, solar radiation information was excluded due to a high frequency of missing values. There were 2898 farms that met all criteria, with a total of 35,392 records spanning from May to July covering the 5-year period. The descriptive statistics of the dataset are provided in Table 1. The daily averages of milk composition and THI across the years are reported in Supplementary Figure S4. Following the findings of Peana et al. [16] on Sarda breeds, the milk yield experienced a significant drop for THI_MAX_ greater than 68. Thus, indicators of heat stress measured indirectly by their detrimental effect on milk composition (THI onsets) were considered for THI_MAX_ > 68 (2 class: 1 = Low, 2 = High). Potential cold stress effects on milk composition were not considered in our study since our dataset structure was not suitable for studying both the cold and heat effects: THI records were available from April to November, but very few bulk milk records were recorded after July.

**Figure 1:**
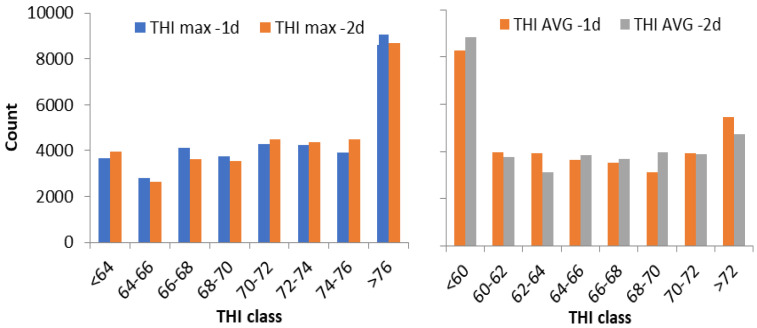
Distribution of records across the different classes of maximum THI (THI_MAX_, on the **left**) and average THI (THI_AVG_,on the **right**).

The THI values 1 and 2 days before milk sampling were grouped into discrete classes according to the distribution of THI and the literature [34]. The THI was categorized into 8 THI_MAX_ classes (1: <64; 2: 64–66; 3: 66–68; …; 8: >76) and 8 THI_AVG_ classes (1: <60; 2: 60–62; 3: 62–64; …; 8: >72); their distributions are reported in Figure 1.

### 2.3. Statistical Analysis

#### 2.3.1. Effect of THI on Bulk Milk Composition

Bulk milk composition traits were analyzed with the following linear mixed model implemented by using the PROC MIXED of SAS (SAS Institute, Cary, NC, USA, 2016):

(2) $$y_{\mathrm{ijkl}}{=\mathrm{mo}(\mathrm{year})}_{i(j)}{+\mathrm{Fs}}_{k}+\mathrm{Fs}\;\times {\mathrm{THI}}_{(k\times l)}{+\mathrm{THI}(\mathrm{year})}_{k(j)}{+\mathrm{THI}(\mathrm{mo})}_{k(i)}{+\mathrm{Flock}(\mathrm{year})}_{l(j)}{+e}_{\mathrm{ijkl}}$$

where *y* is the analyzed milk quality trait (see Table 1) and

- mo(year) is the fixed effect of month (3 levels: May, June and July) nested within year (5 years were considered);
- Fs is the fixed effects of the flock size (3 levels: ≤100, 100–300, >300 heads);
- Fs × THI is the interaction between Fs and THI (8 classes, see Figure 1);
- THI(year) and THI(mo) are the fixed effects of THI nested within year and month, respectively;
- *Flock(year)* and *e* are the random effects of Flock nested within year (6996 levels) and the random residuals distributed, ~N(0, Iσ^2^_Flock_) and ~N(0, Iσ^2^_e_), respectively.

The variation sources related to year, season and THI were treated as fixed effects since the limited number of levels and the possibility of statistical testing (type III sum of squares) contrasting the least square means of different THI– and year–time classes. To account for the farm heterogeneity, the milk quality traits were studied by modeling the flock as a random effect. This was due to the objective difficulty of cross-classifying in meaningful levels all possible combinations of farm management types and other environmental confounding. The percentage of variance explained by flock was calculated as the ratio of σ^2^_Flock_/(σ^2^_Flock_ + σ^2^_e_), where σ^2^_Flock_ and σ^2^_e_ are the flock and the residual variances, respectively. A statistical model of Equation (2) was run both using THI_MAX_ and THI_AVG_ (either one day (−1 d) or two days (−2 d) before milk collection).

The least-square means of milk composition traits for all cross-classified fixed effects were computed and Tukey adjustment was used to correct for post hoc multiple comparisons.

#### 2.3.2. Effects of Multivariate Meteorological Index (MMI) on Bulk Milk Composition

A second approach used multivariate factor analysis (MFA). In our study, the MFA was applied to decompose the correlation matrix of meteorological variables (temperature, relative humidity, wind speed and rainfall) and identify potential latent variables. The measure of sampling adequacy (MSA) of Kaiser was computed to assess the suitability of data to latent factors sought. The factorial model (Equation (3)) used was

(3) $$R=BB^{\prime }+\Psi$$

where **R** is the original correlation matrix and **B** contains the estimates of loading coefficients (b_ij_), i.e., the correlation between original variables and latent factors. The off-diagonal elements of **BB′** provide an approximation of the original correlation matrix, whereas the diagonal elements of **BB′** are the estimates of commonality, i.e., the variance shared by the original variables and explained by the common latent factors. The diagonal matrix **Ψ** brings on the diagonal the uniqueness of each variable (i.e., the part of the variance that each original meteorological variable does not share with the other variables [35]). In our case, the estimates of b_ij_ allowed us to decompose the set of meteorological variables (**y_1_** … **y_p_**) into linear combinations of m (where m < p) latent factors (**f_1_ … f_m_**) plus the residuals (**e**), as described in Equation (4):

(4) $$y_{i}={\;b}_{ij}f_{1}+\ldots +{\;b}_{\mathrm{im}}f_{m}+{\;e}_{i}$$

The latent factors explaining at least 70% of the original variance were retained for further analysis. The varimax rotation of loading coefficients was chosen to simplify the interpretation of the correlation structure. MFA was carried out by implementing the PROC FACTOR of SAS (SAS Institute, Cary, NC, USA). The latent factors were then analyzed with the aim of interpreting their meanings. The latent factors were categorized into 6 discrete classes according to their distribution.

A correlation analysis of THI and latent factors was conducted. The comparisons of THI and multivariate indexes were carried out, including the factor scores extracted by the MFA in the same linear mixed model of Equation (2).

## 3. Results

The weekly averages of milk composition (lactose, fat, protein and casein contents) according to the THI classification (≤68 or >68) were reported in Supplementary Figures S5 and S6. The results of statistical analyses of the effect of climatic conditions (THI or MMI) on bulk milk quality are reported in the following paragraphs.

### 3.1. Effect of THI on Bulk Milk Composition

Table 2 shows the percentage of the trait variances explained by the random effect of the flock (r^2^_Flock_) for each milk composition trait. The flock effect explained 32% (fat and lactose) to 64.4% (log_10_SCC) of the total variance. These estimates indicated that a moderate to large part of bulk milk quality variation was due to environmental and farm management conditions.

All the fixed effects analyzed—month (year), flock size, flock size × THI, THI (year) and THI (month)—were highly significant (*p* < 0.001) for all milk composition traits, with the exception of log_10_SCC (Table 3). For the latter variable, the fixed effects of flock size and its interaction with THI were not statistically significant.

**Figure 2:**
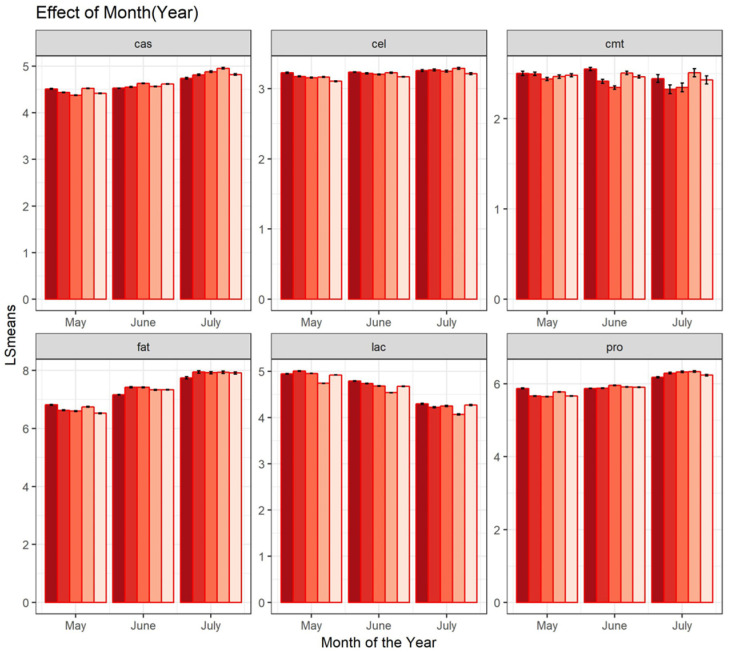
Effect of month nested within year for the 6 analyzed traits (cas = casein%; cel = log10SCC, cmt = total bacterial count, fat = fat%, lac = lactose%, pro = protein%); bars with red color gradient respectively represent a different sampling year. Dark red: first year of sampling; light pink: fifth year of sampling.

The effect of the month of milk collection nested within year had a clear influence on milk composition. In the first year, fat content increased from 6.81% to 7.74% from May to June, protein content increased from 5.87% to 6.17%, casein increased from 4.51% to 4.73%, lactose decreased from 4.94% to 4.30%, the log_10_TBC decreased from 2.52 to 2.48 and log_10_SCC slightly increased (from 3.23 to 3.26). Although these changes differed over the years, the seasonal pattern was confirmed for all bulk milk variables, with the exception of log_10_TBC, which was more erratic. (Figure 2).

**Figure 3:**
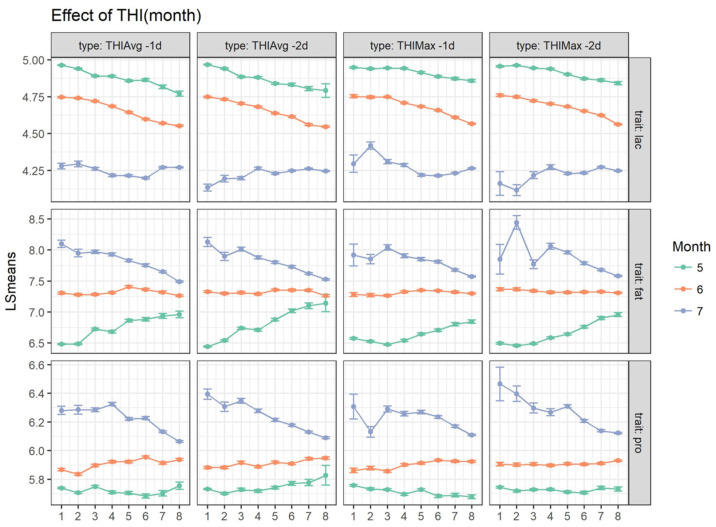
Effects of THI class (on x-axes) nested with month of the year on milk fat composition (lac = lactose%, fat = fat% and pro = protein%). THI_MAX_: **1** ≤ 64, **2**: [64–66], …, **8**: >78; THI_AVG_: **1** ≤ 60, **2**: [60–62], …, **8**: >72.

The effect of THI nested within the month of the year on milk traits showed a similar pattern across different THI measures (THI_AVG_ and THI_MAX_) at −1 d and −2 d before milk collection (Figure 3 and Supplementary Figure S7). The lactose content experienced an evident reduction from May to July (5 in May, 4.7 in June, 4.3 in July), but the pattern in July was not as regular as in the previous months. From May to July, the fat and protein percentages respectively increased from 6.5 to 8.1 and 5.7 to 6.3. In May, fat and protein increased as THI_AVG_ rose; in June, their percentages were stable; whilst, in July, they experienced a drop at higher THI values. Slightly different trends in the milk quality were observed when the THI_MAX_ was considered. With regards to milk fat content in May, +0.3% (THI_AVG_ −2d) and +0.4% (THI_MAX_ −2d) were found at increasing THI values (>70 to >76). In June, no relevant changes were observed, with values remaining almost constant. In July, a drop in the fat content was found as THI values increased (−0.4% and −0.5% for THI_AVG_ −2d and THI_MAX_ −2d, respectively). Similar in trends and magnitudes were the THI effects recorded for protein content. Conversely, the percentage of milk lactose slightly decreased with THI_MAX_ > 72 in May and June and slightly increased in July.

**Figure 4:**
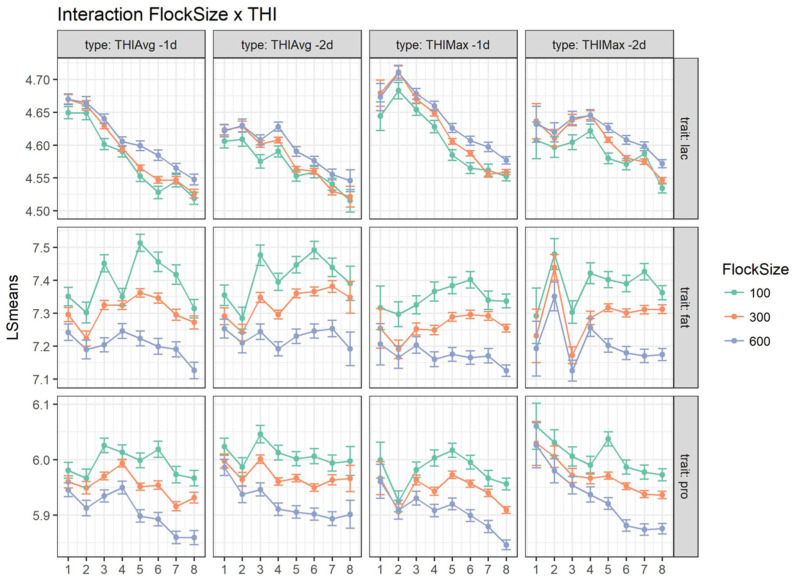
Least square means for milk fat composition (lac = lactose, fat = fat% and pro = protein%), highlighting the interaction THI × flock size. THIMAX: **1** ≤ 64, **2**: [64–66], …, **8**: >78; THIAVG: **1** ≤ 60, **2**: [60–62], …, **8**: >72.

The interaction between flock size and THI (*p*-value < 0.001) is analyzed in Figure 4. Although a general significant effect of the number of animals per flock was observed, only a slight difference among classes of flock dimensions existed, especially for lactose. A decreasing trend for bulk milk protein was observed for higher THI_MAX_ classes −2d before sampling (from 62 to 74), but this decline was minor for bulk milk of small flocks, whilst it was greater for larger farms. Bulk milk fat contents seemed to follow similar patterns across flock sizes, with no clear pattern detected.

### 3.2. Effects of Multivariate Meteorological Index (MMI) on Bulk Milk Composition

The monthly averages of meteorological variables recorded one day before the milk samplings are reported in Table 4 (see also Supplementary Figure S3). The wind speed and rainfall are provided in addition to temperature and humidity.

The pair-wise Pearson correlations of meteorological variables ranged from moderate to high values, with some exceptions (wind speed against all the others). The meteorological variables are clustered into different groups. Temperatures were negatively associated with relative humidity and rainfall. Moderate to high Pearson correlations were observed when the same meteorological variable was recorded the day before or 2 days before milk sampling (Table 5). All these correlations had the largest magnitude when coupling maximum and average values. Finally, pair-wise partial correlations (below the diagonal in Table 5) experienced a substantial drop in magnitude compared with the Pearson correlations (above the diagonal in Table 5), often obtaining values close to zero. This was also confirmed by Kaiser MSA (MSA = 0.71) and suggested the presence of a latent correlation structure.

The factor analysis highlighted two factors able to explain about 70% of the variability. Both were correlated with some of the original variables defining a simple pattern, as reported in Table 6. In particular, factor 1, which explained half of the original variance, was positively correlated with temperature. The loading coefficients of factor 1 were higher (~0.90) for average temperature than for minimum temperature (0.68–0.75). Factor 1 negatively correlated with rainfall and relative humidity, but it was not associated with wind speed.

**Figure 5:**
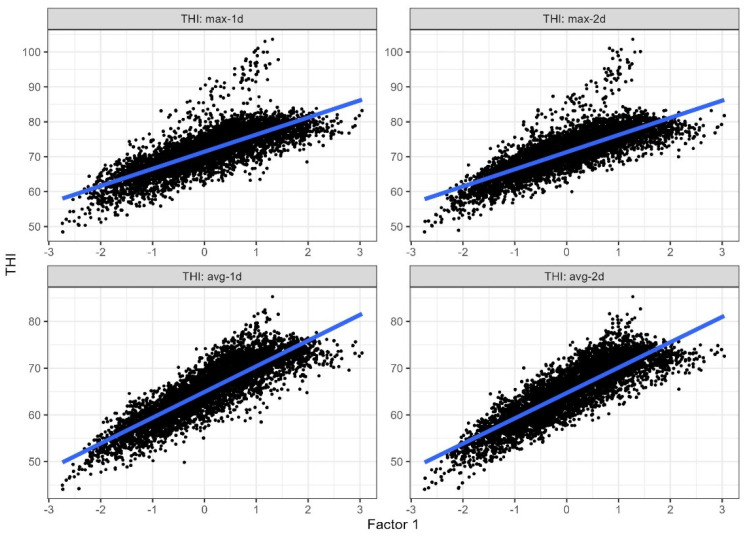
Linear regression between MMI (Factor 1) on the x-axes and THI values on the y-axes (max and average) recorded −1 or −2 days before milk sampling.

The first extracted factor could be described as a THI-like index that we defined as multivariate meteorological index (MMI). The second factor had moderate to high correlations with wind speed and negligible coefficients with all the other variables. In particular, the second factor was neglected due to the low percentage of variance explained and its association with wind speed only. A good agreement between MMI and THI values was observed for all types of THI considered (adjR^2^ = 0.56, Figure 5). Regardless of the days of recording, the highest correlations were found between MMI and THI_AVG_ (Table 7).

**Figure 6:**
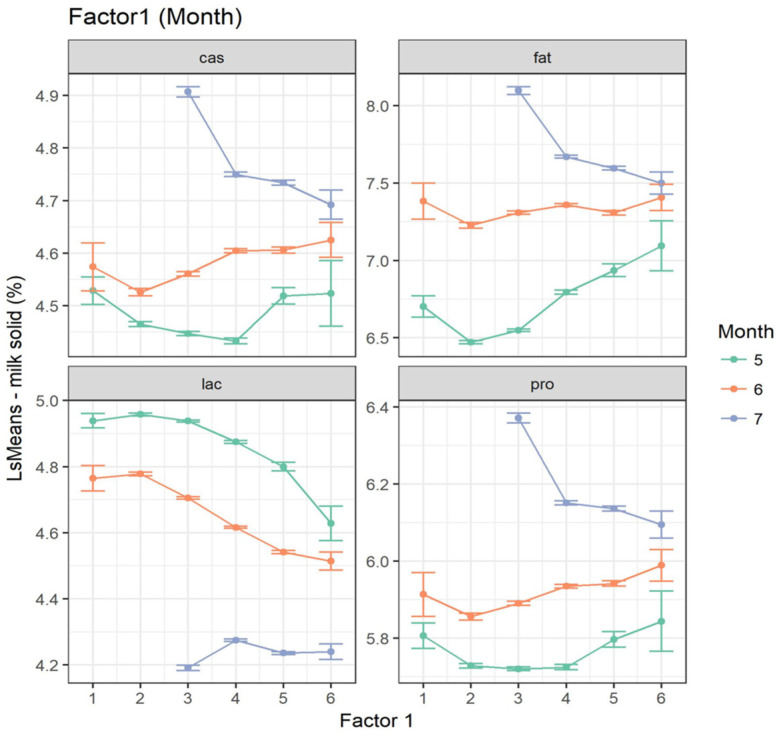
Effect of MMI (Factor 1 (month)) on milk composition (fat = fat%, cas = casein%, lac = lactose%, pro = protein%).

The same linear mixed model used for modeling THI was used to assess the effect of MMI identified by multivariate analysis. At increasing values of MMI, fat, protein and lactose showed the same trend previously observed when analyzing THI classes. All the effects considered were highly significant (*p*-value < 0.001). The least square means for milk traits in different F1 classes are reported in Figure 6.

With reference to milk composition, in May +0.6%, +0.1% and –0.3% were found when passing from MMI class 3 to class 6 for fat, protein and lactose, respectively. In June, no relevant differences were highlighted. Conversely, in July, a decrease in fat (−0.6%) and protein contents (−0.3%) could be observed. An opposite trend was observed for lactose, with average decreases of about −0.2% (−0.1%) from MMI class 3 onward in June and July. Although most of the least square means contrasts were significant, for some classes of MMI, they experienced large standard errors (Figure 6).

## 4. Discussion

Climatic and meteorological conditions are elements of great importance for primary production systems. For the dairy industry, the effects of heat stress can impair the productivity of dairy ruminants raised in a Mediterranean environment. Most of the literature on this topic refers to dairy cattle [15,18,26]. The worsening of sheep and goat reproduction performances under heat stress has been documented [11]. The evaluation of the effects of heat stress on individual dairy ewes’ milk production provides useful insights [1,2,3,4,5] for climate change future scenarios. Our work analyzed milk composition and inferred changes occurring in mid- and late lactation periods using bulk milk and meteorological data. The main limitation of this study is the lack of information on milk yield per ewe, which partially hindered a fair comparison with the literature findings. Due to this fact, we used changes in lactose percentage as an indirect measure of milk yield. The bulk milk fat, protein and casein contents increased as the seasons passed, whereas lactose decreased, as highlighted in Figure 2. This pattern followed the physiological lactation stages. Additionally, the phase of lactation and the weather acting in the same direction made it difficult to distinguish the impact of each on the milk quality, as also observed by other authors for individual milk samples [13,24,36]. The results of the two approaches adopted (THI and MMI) are discussed in the following paragraphs.

### 4.1. Effect of THI on Bulk Milk Composition

The percentage of the variance explained by the flock effects agrees with results in the literature, ascribing a not negligible part of variance to differences among flocks. Research by [37] showed a great influence of flock in explaining variance for test day milk yield, fat, protein and casein percentages in dairy sheep. A similar amount of variance was also found in dairy sheep in other works [38,39]. The overall statistical testing highlighted significant differences for almost all milk composition traits and the considered environmental effects. The effect of the month of the year in our study agrees with the literature [15], suggesting that the relationships between milk composition and weather differed among the three phases of lactation (early, mid-, and late lactation). Somatic cell count did not have a clear trend, remaining nearly constant, whereas the total bacterial count showed higher inter-annual variability in June and July, conversely to what has been observed for dairy cattle, as dairy sheep farming is characterized by the high seasonality of lambing (Figure 2).

The examination of milk quality across THI class and month of recording (Figure 3) showed higher lactose contents under no-stress conditions (THI class < 6) and lower contents for THI > 6. Fat, protein and casein followed an opposite trend. Lactose, fat and protein contents presented different behaviors according to the THI class within the month of the year. For higher THI values, a reduction of milk lactose was observed. However, this happened in the warmest period of the year, which corresponded to the last stage of lactation in dairy ewes [16]. Furthermore, the positive THI effects on the fat and protein percentages could be partially explained by the solid concentration that occurs during the last stage of lactation. This normally coincides with the late spring–early summer period. A similar result was found by [13], who also reported positive correlation coefficients equal to 0.15 and 0.10 for fat and protein content, respectively, as THI increased.

Several studies underpin the effect of THI on milk traits, but their effects vary within species and breed. Furthermore, the systematic measurement of heat stress in sheep remains problematic, particularly because of the overlap with seasonal changes in milk quality due to physiological reasons. This fact limits the possibility to distinguish the effect of the stage of lactation from the effect of heat waves. It was not possible to completely separate the two effects. There was also a lack of information, such as the average lambing periods of the analyzed farms [15]. The possible effects exerted by bio-meteorological factors on milk components might suggest a time correlation between bio-meteorological variables and milk composition that needs to be further disentangled.

The interaction between flock size and THI classes was significant. In our study, farm averages were used and higher fat and protein and lower lactose contents corresponded to smaller flock sizes. The milk composition averages for different flock sizes were small and could be partially ascribed to differences among farming systems [40]. Environmental effects in the literature, such as herd size and management, justify variances that are marginally higher in larger herds compared to smaller ones [32]. In Sardinia, bigger flocks generally use of modern facilities and feeding techniques, which allow for a higher yield. Small flocks are generally kept by family operations and use traditional management and feeding techniques, rearing less animals [40]. According to the literature, we found that in some cases (protein and fat contents), THI exerted a slightly different effect according to the flock dimension considered. The effect of flock size on bulk milk quality was also observed by [41] in goat farming regarding microbiological quality but not milk solid composition.

In our dataset, no information regarding feeding management was available. However, the feeding regime was the environmental factor that highly impacted milk yield and composition, especially the botanical composition of pastures and diet composition (e.g., protein and fiber contents). The availability of pasture in the Mediterranean lowland progressively increases from September after a period of less availability in winter and reaches its peak in April [29]. Further, it drops, corresponding to the lowest availability of grass, in the final part of lactation.

### 4.2. Effects of Multivariate Meteorological Index (MMI) on Bulk Milk Composition

The meteorological variables presented values in agreement with the recent historical series observed in Sardinia (https://www.sardegnaambiente.it/arpas/, accessed on 6 September 2024). It is worth underlining that the impact of humidity on the thermal balance of an animal is species-specific and varies among individuals [42]. Despite this, in Sardinian climate, the effect of humidity was lower than that exerted in a humid climate because the hottest temperature generally corresponded to the lowest relative humidity, without being hindered by evaporative heat losses. In this situation, the low relative humidity corresponded to decreases in the thermal perception, as shown also by the formula of THI max, which combined maximum daily temperature and minimum relative humidity [24].

Heat stress is produced by a combination of several factors including temperature, relative humidity, solar radiation, air movement and precipitation, which can mitigate the total heat load [43]. Our study aimed to characterize a thermal stress index at the farm level and integrate all known meteorological data into a single measure (Table 6 and Figure 5). The use of multivariate approaches allowed us to synthesize complex correlations without imposing specific constraints on the use of the data, extracting new variables able to better describe the behavior. At the same time, the use of THI (maximum, average or minimum) required the selection of the data used (e.g., periods of heat waves, temperatures and humidity to be used, etc.). Multivariate approaches have been recently applied to estimate or classify heat tolerance genotypes in dairy and beef cattle [15,17]. In our study, quality milk data were merged with weather information to develop, by means of multivariate techniques, a representative factor (MMI) that reflected and assessed the potential impact of heat stress. This approach allowed us to neglect the determination of the most appropriate THI function because it simultaneously used all available meteorological information. The components that primarily contributed to the commonalities were the minimum and average temperatures and relative humidity two days before milk sampling. It reflected the large weights of temperatures in the THI, the effect of which was predominant (0.82–0.95 of the variance, depending on the formula used) in the index [42]. When considering the effect of MMI on milk quality traits, similar results of the application of THI were found.

## 5. Conclusions

The effect of THI on milk composition was statistically significant across different bulk milk quality traits (*p* < 0.001). However, the overlapping effect of seasonality needed to be considered. THI exerted a slightly different effect according to the flock dimension considered. The use of multivariate techniques allowed us to merge weather information, developing the representative multivariate factor highly correlated with THI (0.75). The MMI reflected the THI and assessed the potential impact of heat stress using all available information. Temperature positively contributed to MMI, while humidity was negatively correlated to it. This behavior reflected the effect of humidity in decreasing the thermal perception. The inclusion of climatic variables such as temperature, relative humidity, wind speed and rainfall could improve the effects of environmental stressors on dairy performances.

## Figures and Tables

**Table 1:** Descriptive statistics for bulk milk composition traits and bio-meteorological indexes from May to July for 5-year sampling periods.

Variable ^1^	Mean	SD	Min	Max
Fat content, g/100 mL	7.09	0.77	3.5	11.8
Protein, g/100 mL	5.89	0.36	4.2	8.0
Casein, g/100 mL	4.57	0.29	3.1	6.2
Lactose, g/100 mL	4.67	0.35	2.5	5.4
log_10_ TBC, log_10_ total bacterial count	2.48	0.73	0.3	4.2
log_10_ SCC, log_10_ somatic cell count	3.21	0.25	1.4	4.2
THI_MAX_ −1d	71.44	6.06	48.5	103.6
THI_MAX_ −2d	71.32	6.03	48.5	103.6
THI_AVG_ −1d	64.91	6.22	44.1	85.3
THI_AVG_ −2d	64.70	6.21	44.1	85.3

^1^ Total of 35,392 records from 2898 farms; SD: standard deviation.

**Table 2:** Variance explained by flock.

Trait	r^2^_Flock_ (%)
Fat, g/100 mL	32.0
Protein, g/100 mL	41.1
Casein, g/100 mL	41.3
Lactose, g/100 mL	32.5
CMT, log_10_CMT	37.3
SCC, log_10_SCC	64.4

**Table 3:** Significance of fixed effects for log10SCC using different measures of THI.

		log_10_SCC			
Effect		THI_AVG1_	THI_AVG2_	THI_MAX1_	THI_MAX2_
Month (year)	mo(year)	<0.001	<0.001	<0.001	<0.001
Flock size	Fs	0.14	0.22	0.16	0.19
Flock size × THI	Fs × THI	0.11	0.52	0.75	<0.001
THI (year)	THI(year)	<0.001	<0.001	<0.001	<0.001
THI (month)	THI(mo)	<0.001	<0.001	<0.001	<0.001

**Table 4:** Mean and standard deviation (SD) of meteorological values on the day of milk sampling and the day before from May to July.

	May		June		July	
Meteorological Variable	Mean	SD	Mean	SD	Mean	SD
T_AVG_, °C	16.1	3.4	20.6	4.0	23.8	3.1
T_MAX_, °C	22.2	4.7	27.8	5.2	31.4	4.2
T_MIN_, °C	10.1	3.6	13.7	4.1	16.6	3.5
RH_AVG_, %	70.5	14.9	63.4	15.6	55.0	13.3
RH_MAX_, %	91.1	10.4	88.2	13.0	83.9	13.2
RH_MIN_, %	46.8	19.4	37.6	16.6	28.9	12.7
WS_AVG_, m/s	3.1	1.9	2.7	1.3	3.1	1.9
WS_MAX_, m/s	11.8	4.8	10.9	3.1	12.1	4.0
Rainfall, mm	2.1	6.0	0.6	2.7	0.2	1.7

**Table 5:** Pearson correlation (above the diagonal) and partial correlation (below the diagonal) between pairs of meteorological variables recorded 1 or 2 days before milk sampling.

	T_M1_	T_M2_	T_m1_	T_m2_	T_A1_	T_A2_	RH_M1_	RH_M2_	RH_A1_	RH_A2_	RH_m1_	RH_m2_	WS_A1_	WS_A2_	WS_M2_	WS_M2_	R_F1_	R_F2_
**T_M1_**		0.79	0.67	0.54	0.95	0.76	−0.42	−0.34	−0.70	−0.53	−0.71	−0.52	−0.22	−0.20	−0.09	−0.09	−0.34	−0.28
**T_M2_**	0.33		0.67	0.66	0.81	0.95	−0.32	−0.41	−0.51	−0.70	−0.50	−0.70	−0.06	−0.20	0.05	−0.06	−0.21	−0.35
**T_m1_**	−0.49	0.00		0.81	0.84	0.79	−0.47	−0.41	−0.44	−0.44	−0.30	−0.35	0.05	−0.07	0.09	−0.03	−0.09	−0.18
**T_m1_**	0.02	−0.58	0.27		0.69	0.84	−0.34	−0.47	−0.34	−0.45	−0.24	−0.30	0.04	0.06	0.09	0.10	−0.08	−0.11
**T_A1_**	0.87	−0.21	0.74	−0.14		0.84	−0.49	−0.40	−0.67	−0.54	−0.61	−0.50	−0.13	−0.17	−0.03	−0.08	−0.29	−0.27
**T_A1_**	−0.22	0.88	−0.08	0.80	0.26		−0.36	−0.48	−0.50	−0.68	−0.45	−0.60	−0.03	−0.12	0.06	−0.01	−0.18	−0.30
**RH_M1_**	−0.05	−0.01	−0.26	0.06	0.16	−0.03		0.55	0.75	0.47	0.43	0.29	−0.17	−0.06	−0.14	−0.06	0.19	0.14
**RH_M2_**	−0.02	−0.12	0.04	−0.28	−0.03	0.22	0.23		0.47	0.75	0.29	0.42	−0.11	−0.18	−0.11	−0.15	0.12	0.19
**RH_m1_**	0.21	−0.06	0.28	−0.09	−0.36	0.15	0.76	−0.09		0.64	0.87	0.58	−0.03	0.03	−0.05	0.00	0.39	0.25
**RH_m2_**	−0.09	0.31	−0.05	0.36	0.15	−0.45	−0.10	0.76	0.21		0.56	0.86	−0.05	−0.05	−0.07	−0.07	0.18	0.39
**RH_a1_**	−0.43	0.20	−0.10	0.00	0.33	−0.14	−0.47	−0.02	0.77	−0.14		0.63	0.12	0.12	0.08	0.08	0.45	0.27
**RH_a2_**	0.23	−0.48	−0.05	−0.16	−0.12	0.37	−0.03	−0.48	−0.08	0.77	0.28		0.06	0.09	0.02	0.04	0.19	0.44
**WS_a1_**	−0.21	0.15	0.04	0.01	0.09	−0.08	−0.08	0.06	−0.01	−0.05	−0.06	0.10		0.48	0.77	0.31	0.07	0.09
**WS_a2_**	0.15	−0.16	0.03	0.07	−0.10	0.03	0.07	−0.03	−0.03	−0.06	0.06	−0.03	0.50		0.33	0.73	0.13	0.09
**WS_M1_**	0.09	0.00	0.10	−0.07	−0.11	0.05	0.12	−0.04	−0.12	0.04	0.09	0.02	0.74	−0.32		0.41	0.18	0.12
**WS_M2_**	−0.01	0.07	−0.09	0.10	0.02	−0.05	−0.06	0.04	0.01	−0.02	0.03	0.01	−0.35	0.70	0.43		0.17	0.18
**R_F1_**	0.08	−0.05	0.13	0.00	−0.10	0.02	−0.04	0.09	0.02	−0.05	0.18	−0.07	−0.20	0.06	0.23	−0.01		0.19
**R_F2_**	−0.04	0.05	−0.03	0.12	0.03	−0.09	0.03	−0.01	−0.02	0.01	−0.04	0.14	0.04	−0.15	0.01	0.19	0.10	

T_M1(2)_, T_m1(2)_ and T_a1(2)_ = Maximum (M), minimum (m) and average (a) temperature (°C) 1 or 2 (days) before milk sampling; RH = Relative Humidity (%); WS = wind Speed (m/s); RF = rainfall (mm).

**Table 6:** Factorial scheme. Loading coefficients for varimax rotation (in bold: loading coefficients > 0.30).

Variable	Factor 1	Factor 2	Commonality
T_AVG_ −1d (°C)	**0.90**	−0.16	0.80
T_AVG_ −2d (°C)	**0.89**	−0.01	0.76
T_MAX_ −2d (°C)	**0.87**	−0.10	0.56
T_MAX_ −1d (°C)	**0.86**	−0.25	0.49
T_MIN_ −1d (°C)	**0.75**	0.05	0.84
T_MIN_ −2d (°C)	**0.68**	0.14	0.80
Rfall −1d (mm)	−0.29	0.22	0.36
Rfall −2d (mm)	**−0.35**	0.17	0.41
RH_MAX_ −1d (%)	**−0.57**	−0.18	0.59
RH_MAX_ −2d (%)	**−0.59**	−0.26	0.64
RH_MIN_ −1d (%)	**−0.68**	0.19	0.49
RH_MIN_ −2d (%)	**−0.70**	0.08	0.49
RH_AVG_ −1d (%)	**−0.77**	0.01	0.53
RH_AVG_ −2d (%)	**−0.79**	−0.11	0.51
WS_AVG_ −1d (ms^−1^)	0.00	**0.73**	0.48
WS_AVG_ −2d (ms^−1^)	−0.07	**0.71**	0.43
WS_MAX_ −1d (ms^−1^)	0.06	**0.69**	0.13
WS_MAX_ −2d (ms^−1^)	0.00	**0.66**	0.15
**Var Explained %**	**51.9**	**17.4**	

**Table 7:** Pairwise Spearman rank correlation between THI and MMI.

Class	THI_MAX_ −1d	THI_MAX_ −2d	THI_AVG_ −1d	THI_AVG_ −2d
MMI (Factor 1)	0.75	0.75	0.81	0.80
THI_MAX_ −1d		0.79	0.93	0.77
THI_MAX_ −2d			0.81	0.94
THI_AVG_ −1d				0.84

## Data Availability

Climatic and farm data that support the findings of this study are available from the corresponding author [G.G.] upon reasonable request.

## Supplementary Materials

The following supporting information can be downloaded at https://www.mdpi.com/article/10.3390/ani15040533/s1: Supplementary Table S1. Location and altitude of Sardinian meteorological network and the number of farms associated with each station; Supplemental Figure S1. Distribution of farm records (monthly milk samples); Supplemental Figure S2. Network of meteorological stations in Sardinia; Supplementary Figure S3. Historical temperature elaborated upon by ARPAS, which publishes climatic maps of Sardinia Island based on historical data; Supplemental Figure S4. Daily averages for milk composition (fat, protein, casein and lactose%) of the whole dataset (on the secondary right axis) and daily THI from three days before (−3 d) to (0 d) milk sample collection (top) and raw data and smoothing curves for milk composition (on the secondary right axis) and THI (on the left axis) from May to August (bottom); Supplemental Figure S5. Description of the relationship between milk traits during the days of the year according to THI class (1 ≤ 68 and 2 > 68); Supplemental Figure S6. Description of the relationship between milk traits and THI of the days before milk sample collection (1 ≤ 68 and 2 > 68); Supplementary Figure S7. Description of the relationship between bulk milk composition and THI level of the 2 days before milk sample collection (red 1 lines ≤ 68 and blue lines 2 > 68) in May, June and July.
