# Supplementary material for: Relationship Between Bio-Climatic and Milk Composition Data of Dairy Sheep Farms: Comparison Between THI and Multivariate Weather Index
Source: Animals (Basel). 2025 Feb 13;15(4):533. doi: 10.3390/ani15040533 (PMC11851843; doi:10.3390/ani15040533)
Supplement: Supplementary file 1 [file animals-15-00533-s001.zip › animals-3350649-supplementary.pdf]

Supplementary Materials

Supplementary table S1 Location and altitude of Sardinian meteorological network, number of farms associated to each Station

Supplemental Figure S1. Distribution of number of farm records (monthly milk samples)

Supplemental Figure S2. Network of meteorological stations in Sardinia (Picture from ARPA Sardegna – http://www.sar.sardegna.it/documentazione/strumenti/retestazioni.asp)

Supplementary Figure S3. Historical temperature elaborated by ARPAS which publish climatic map of Sardinia Island based on historical data (http://www.sar.sardegna.it/)

Supplemental Figure S4. top) Daily averages for milk composition (fat, protein, casein and lactose%) of the whole datasets (on the secondary right axis) and daily THI from three days before (-3 d) to (0 d) from the milk sample collection; bottom) raw data and smoothing curves for milk composition (on the secondary right axis) and THI (on the left axis) from May to August

Supplemental Figure S5. Description of relationship between milk traits during the days of the year according to THI class (1 ≤68 and 2>68)

Supplemental Figure S6. Description of relationship between milk traits and THI of the days before milk sample collection (1 ≤68 and 2>68)

Supplementary Figure S7. Description of relationship between bulk milk composition and THI level of the 2 days before milk sample collection (red 1 lines ≤68 and blue lines 2>68) on May, June and July.

Supplementary table S1 Location and altitude of Sardinian meteorological network, number of farms associated to each Station

Meteo Station Information[1]			
**Station (n=60)**	**Latitude (UTM n)**	**Alt[2]**	**Tot[4]**
GIAVE	4479237	410	236
CHIARAMONTI	4508715	368	212
OLMEDO	4501382	31	194
SIURGUSDONIG.	4386183	414	175
BENETUTTI	4475850	284	169
BITTI	4480550	782	149
CHILIVANIUCEA	4495583	290	141
USINI MOBILE	4500817	197	125
PUTIFIGARI	4488593	422	120
SINISCOLA	4492740	14	119
BERCHIDDA	4514900	276	117
OTTANA	4453749	160	116
MASAINAS	4322389	57	102
GONNOSFANAD.	4373392	146	100
OROSEI	4468859	26	96
GHILARZA	4441089	293	92
ILLORAI	4469946	878	92
MODOLO	4459270	241	90
BONNANARO	4490098	345	90
GAVOI	4446812	835	89
NURALLAO	4406310	380	87
MACOMER	4457378	665	85
S. MONTIFERRO	4453110	491	80
SAN TEODORO	4513069	15	80
DORGALI MOB	4456432	156	78
ALLAI	4422993	60	78
SARDARA	4383164	189	62
VALLEDORIA	4532148	2	62
IGLESIAS	4352022	208	58
OLIENA	4457432	132	53
GUASILA	4375315	246	49
SADALI	4407653	780	48
ZEDDIANI UCEA	4426664	10	47
VILLACIDRO	4366984	130	44
ATZARA	4426815	620	44
SASSARI S.A.R.	4509934	150	38
ORGOSOLO	4450014	290	36
SILIQUA	4350085	0	34
MEANA SARDO	4422210	0	34
MURAVERA	4363009	2	33
P. ARBOREA	4412260	20	33
DORGALI FILIT.	4469983	85	28
NUORO	4465479	488	28
SORSO	4519874	50	25
SAMASSI	4375110	89	24
BARISARDO	4411311	51	24
ARBOREA	4412260	20	22
JERZU	4404776	47	21
DOLIANOVA	4359890	165	20
MILIS	4434476	97	20
OZIERI	4490655	238	19
UTA	4343369	20	15
LURAS	4531649	488	12
DECIMOMANNU	4351919	29	9
STINTINO	4532139	35	8
ARZACHEN MOB	4545082	120	8
MONASTIR MOB	4355655	96	5
VILLANOVA ST.	4423612	813	4
ARITZO	4422227	879	3
ARZACHENA	4545082	83	1

[1] In the first column the municipality where the Meteorological Station are located. [2]Altitude, Meter above the sea level. [3]The number of farms for which both meteorological and milk production data were available.

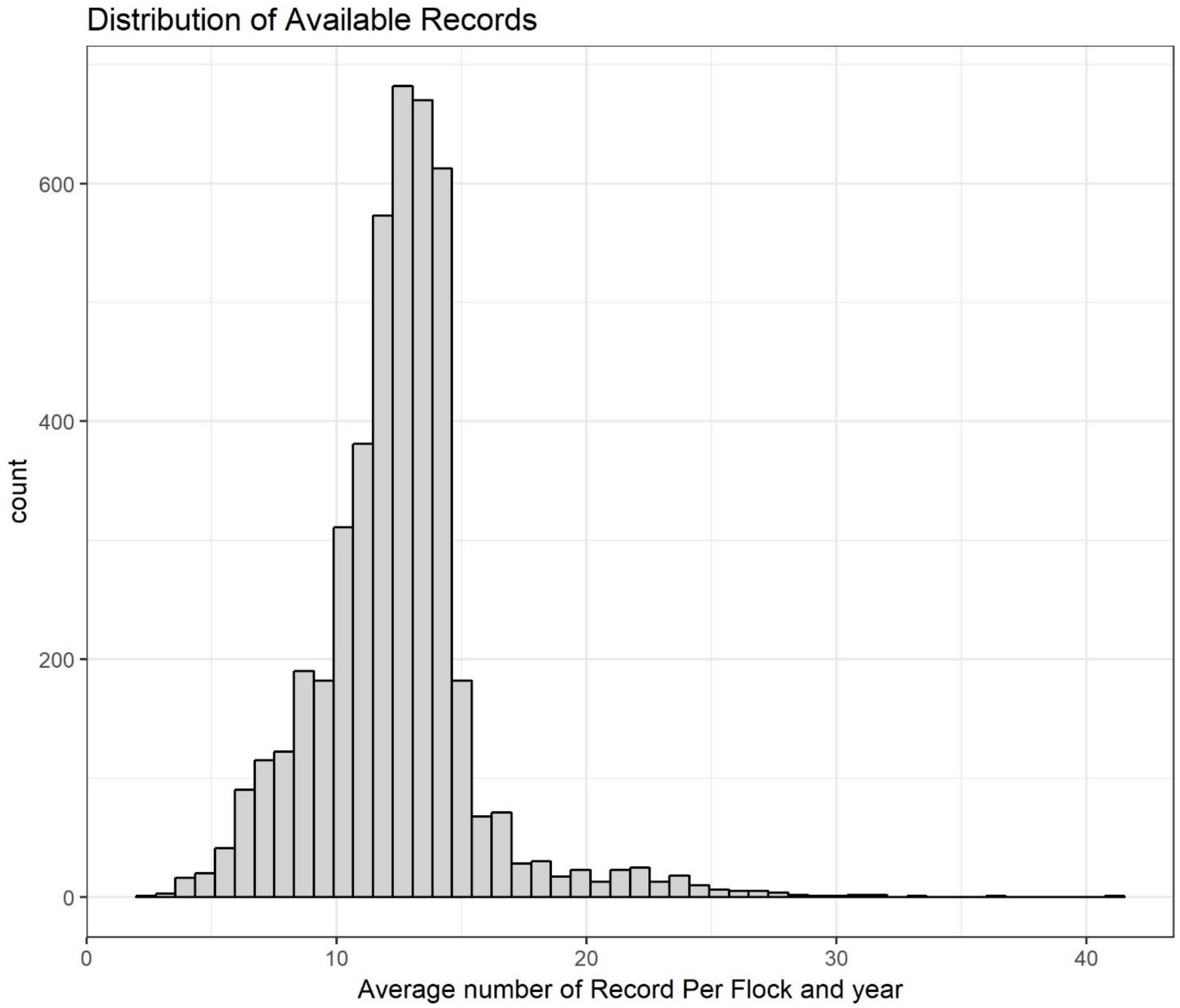

Supplemental Figure S1. Distribution of number of farm records (monthly milk samples)

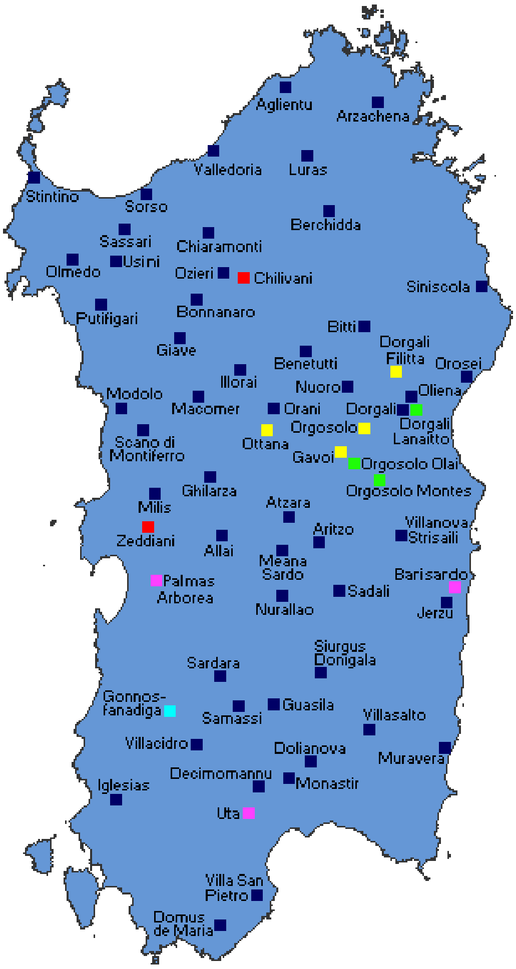

Supplemental Figure S2. Network of meteorological stations in Sardinia (Picture from ARPA Sardegna – http://www.sar.sardegna.it/documentazione/strumenti/retestazioni.asp)

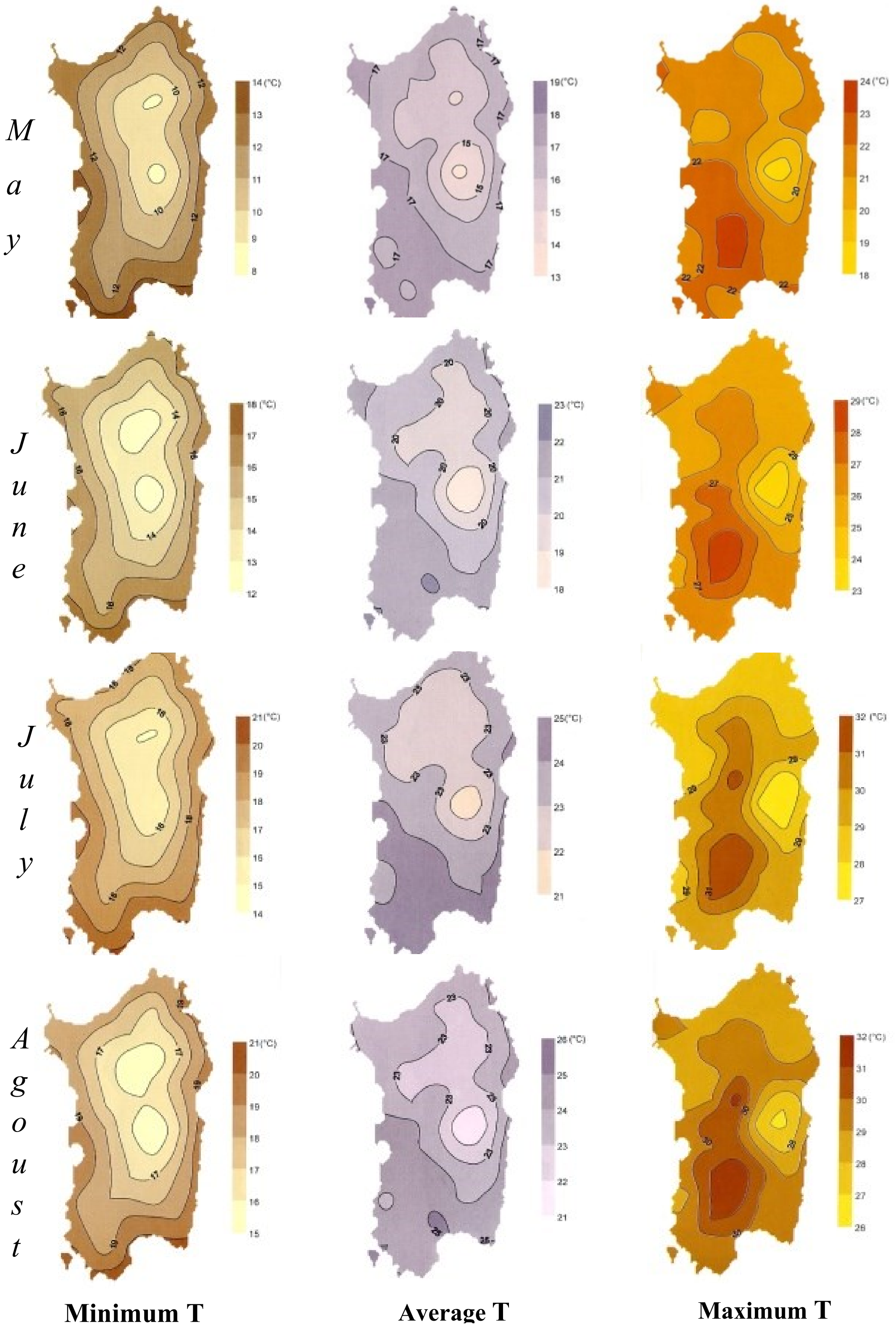

Supplemental

Supplementary Figure S3 Historical temperature elaborated by ARPAS which publish climatic map of Sardinia Island based on historical data (http://www.sar.sardegna.it/)

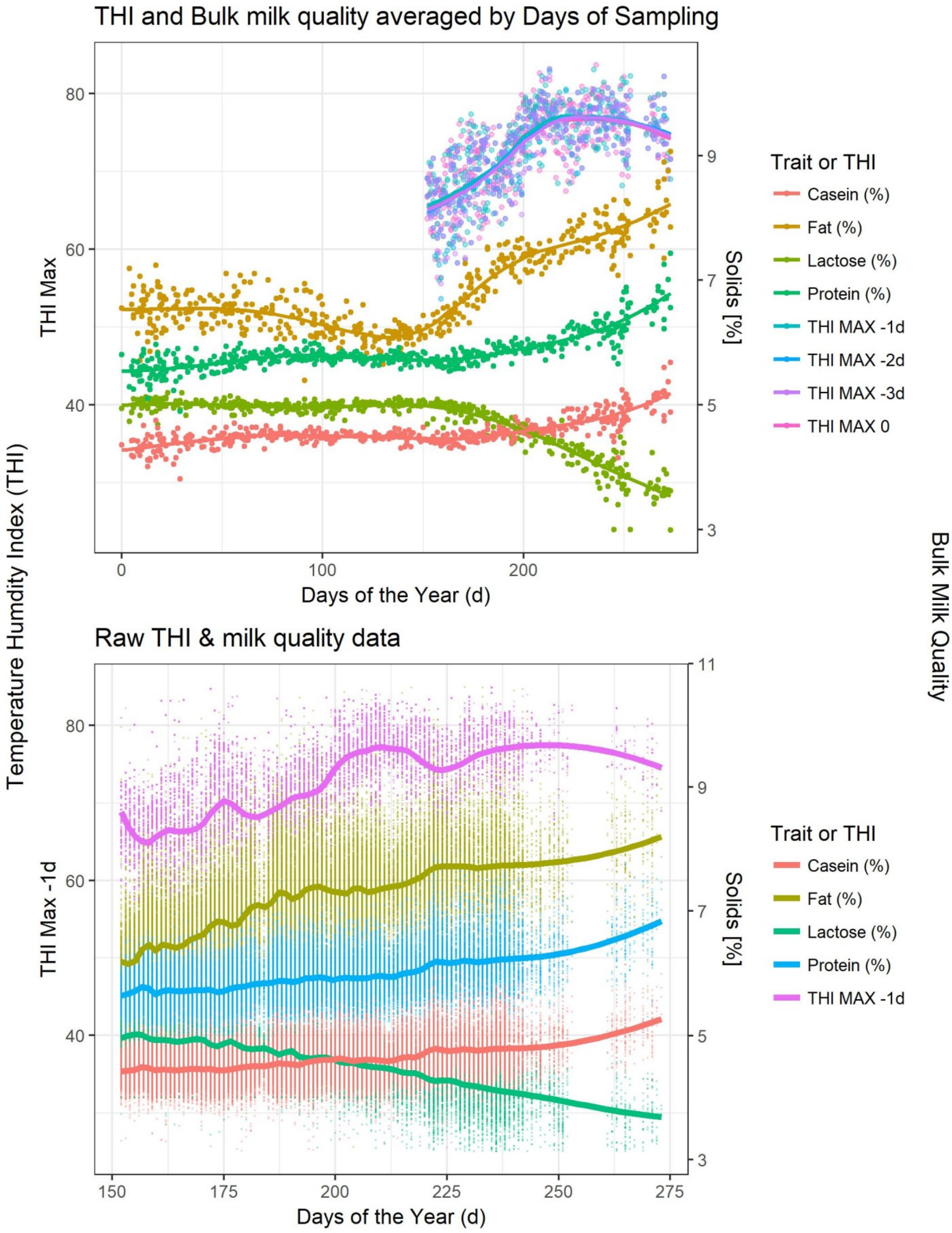

Supplemental Figure S4 top) Daily averages for milk composition (fat, protein, casein and lactose%) of the whole datasets (on the secondary right axis) and daily THI from three days before (-3 d) to (0 d) from the milk sample collection; bottom) raw data and smoothing curves for milk composition (on the secondary right axis) and THI (on the left axis) from May to August.

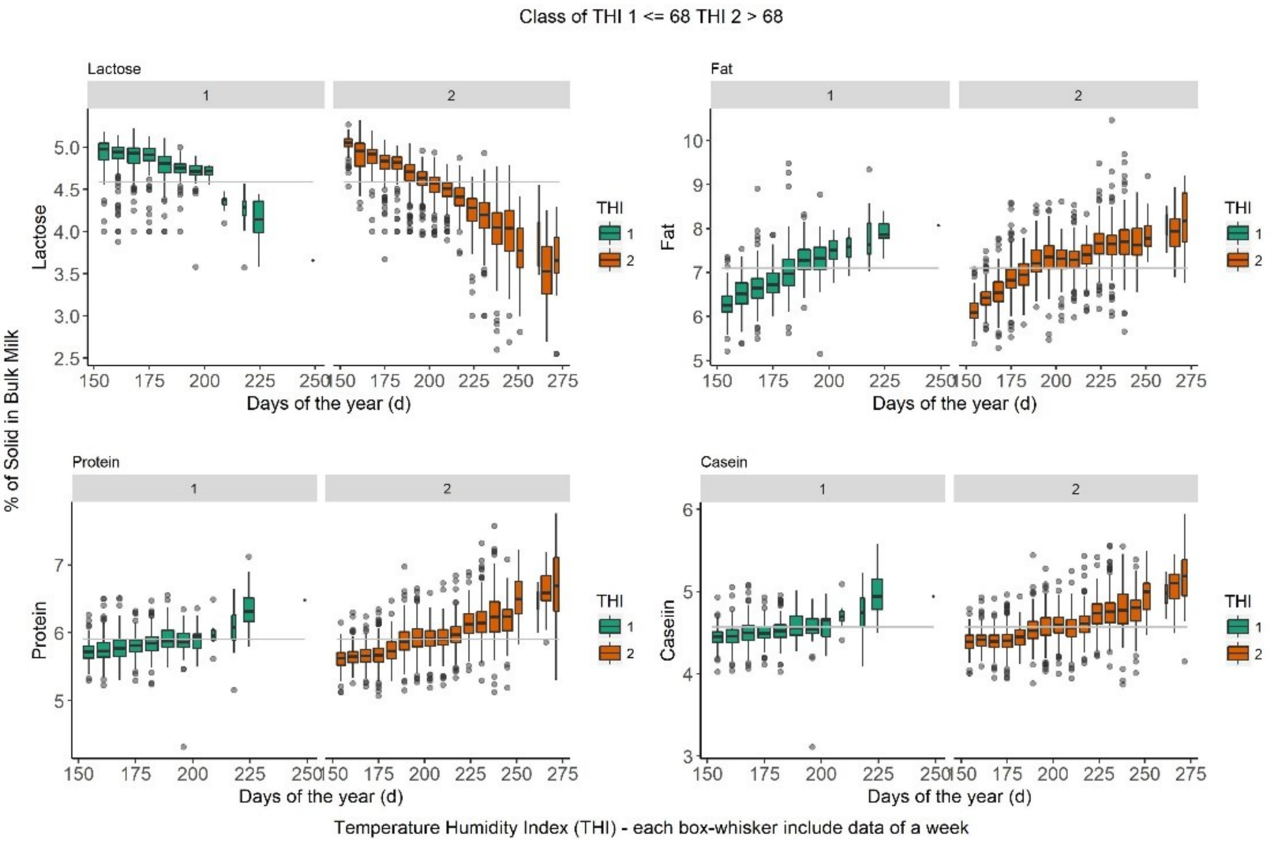

Supplemental Figure S5 Description of relationship between milk traits during the days of the year according to THI class (1 ≤68 and 2>68)

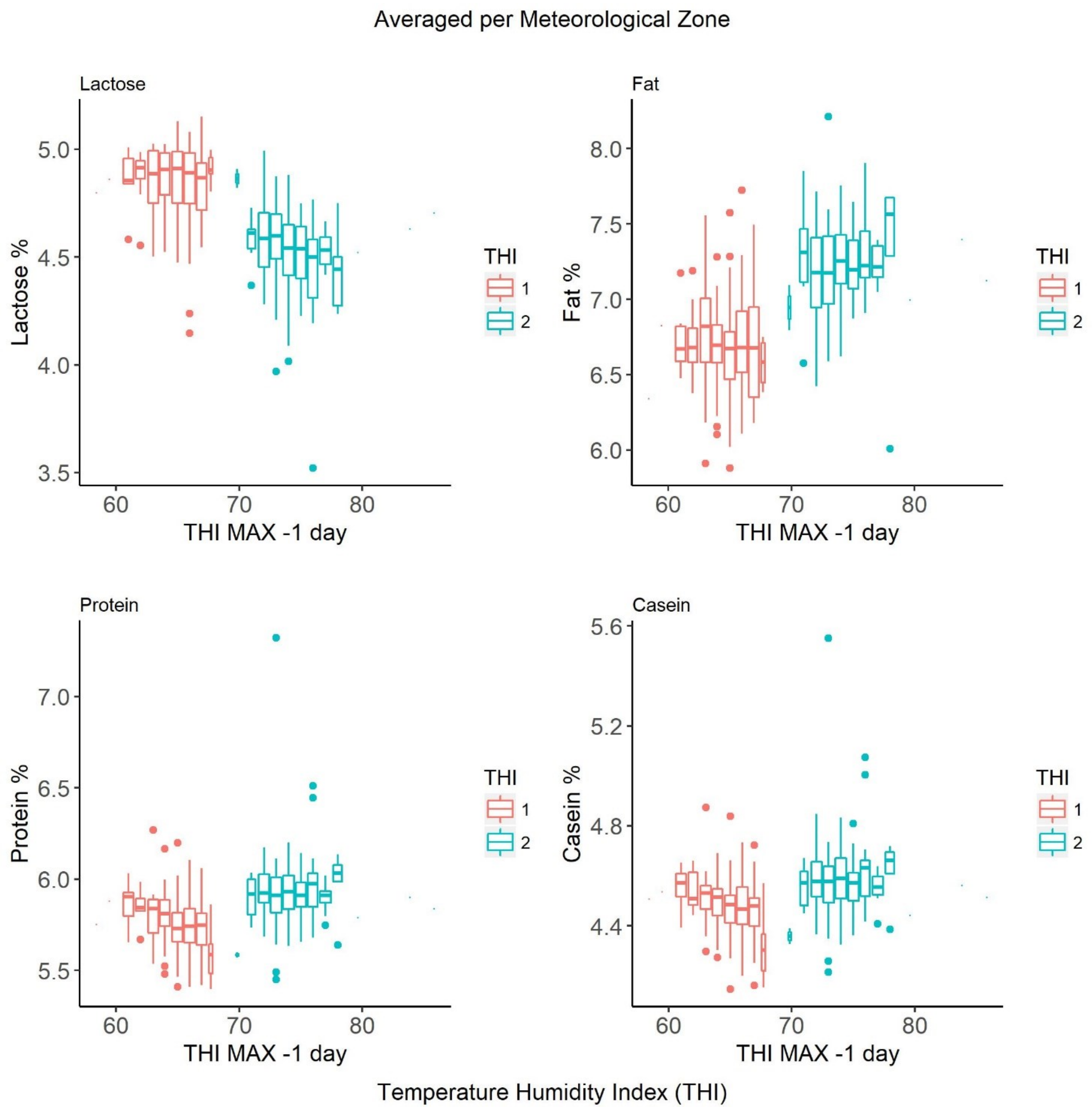

Supplemental Figure S6 Description of relationship between milk traits and THI of the days before milk sample collection (1 ≤68 and 2>68)

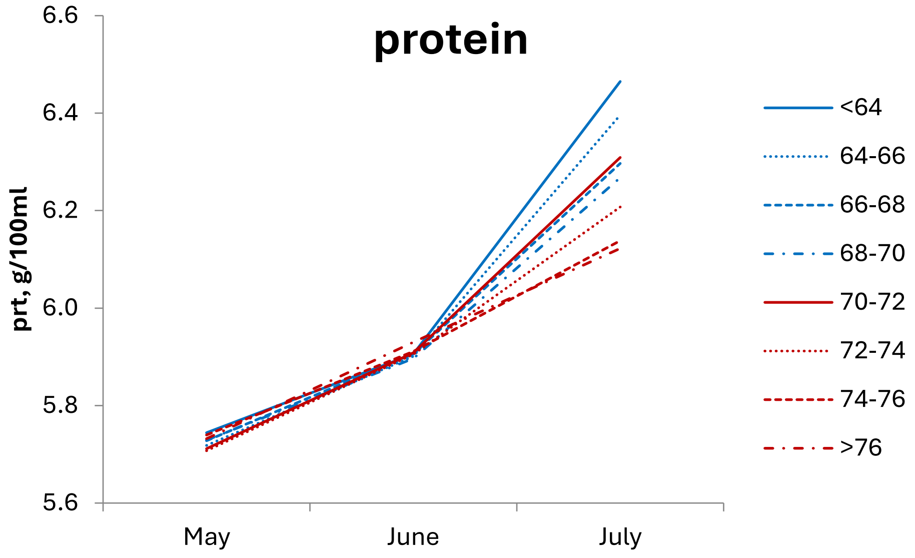
protein
prt, g/100ml
6.6
6.4
6.2
6.0
5.8
5.6
May
June
July
<64
64-66
66-68
68-70
70-72
72-74
74-76
>76

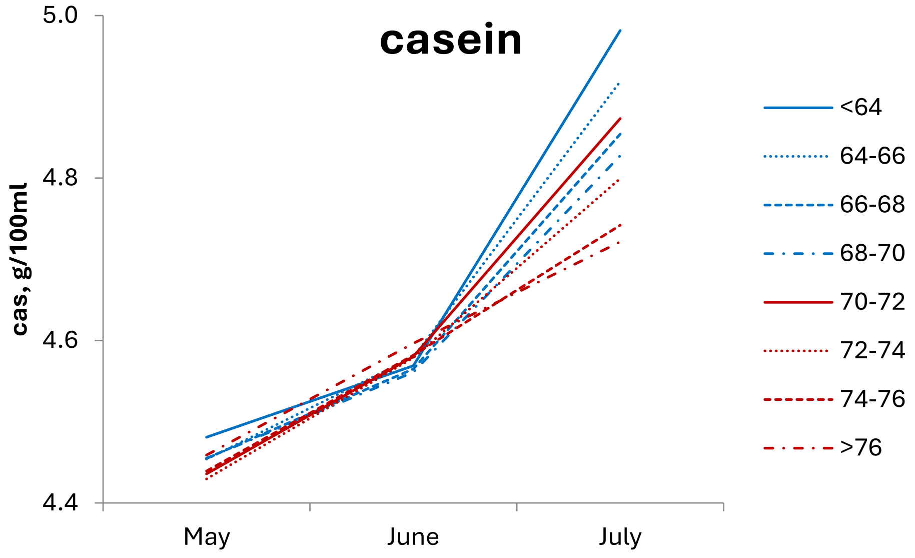
casein
cas, g/100ml
5.0
4.8
4.6
4.4
May
June
July
<64
64-66
66-68
68-70
70-72
72-74
74-76
>76

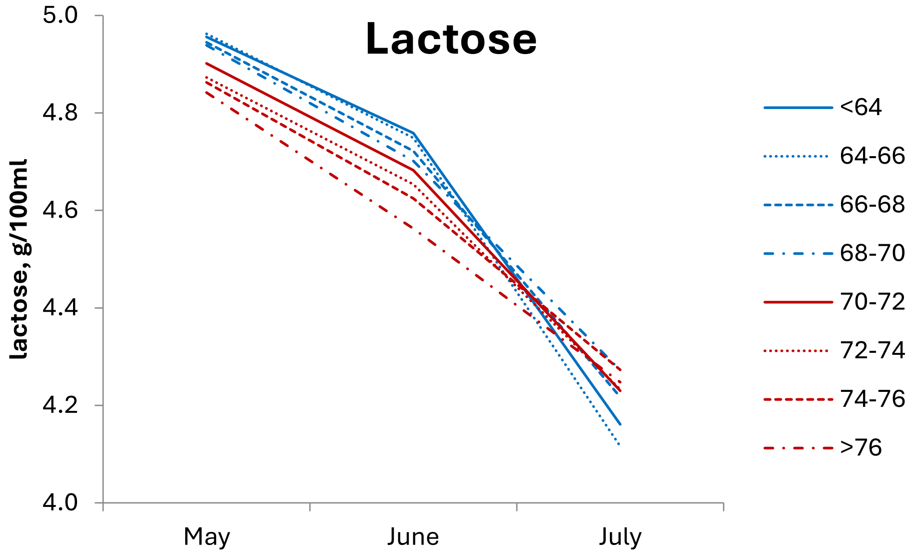
Lactose
lactose, g/100ml
5.0
4.8
4.6
4.4
4.2
4.0
May
June
July
<64
64-66
66-68
68-70
70-72
72-74
74-76
>76

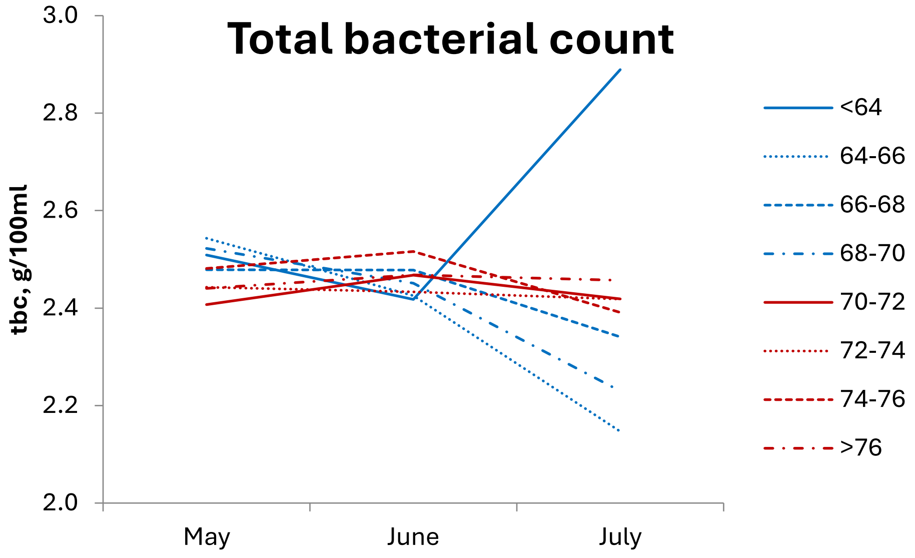

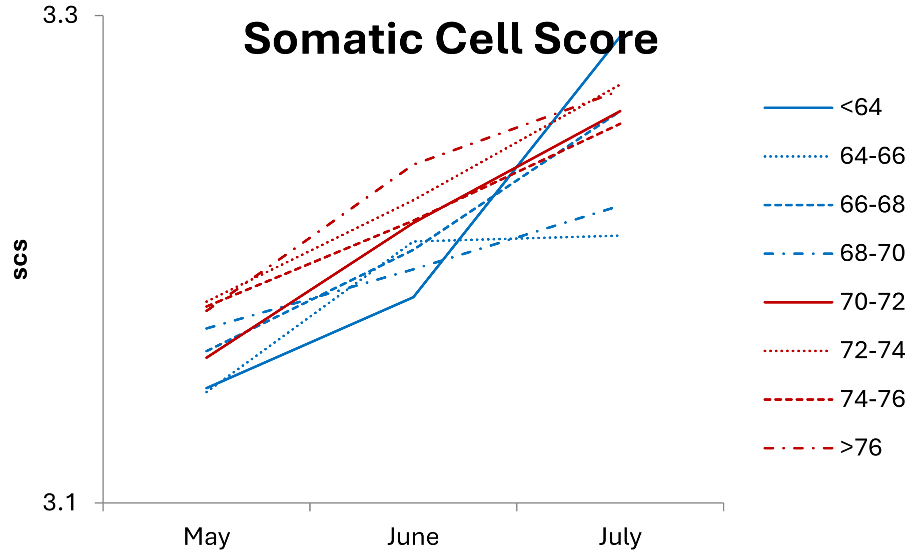

Supplementary Figure S7. Description of relationship between bulk milk composition and THI level of the 2 days before milk sample collection (red 1 lines ≤68 and blue lines 2>68) on May, June and July.
